# Mid and long-term follow-up of 50 pediatric cardiac Chadians operated in France from 2003 to 2012

**DOI:** 10.11604/pamj.2022.42.66.29243

**Published:** 2022-05-24

**Authors:** Neguemadji Ngardig Ngaba, Jean Philippe Lesbre, Namrata Hange, Maria Kezia Lourdes Ligsay Pormento, Molly Sanjay Jain, Manoj Kumar Reddy Somagutta, Vikash Jaiswal, Uzoego Nwakaku Chibuzo, Imteyaz Ahmad Khan, Sana Irfan Khan

**Affiliations:** 1 Centre Hospitalier Universitaire Bon Samaritain de Walia, N´Djamena, Tchad,; 2 Centre Hospitalier Universitaire Amiens-Picardie, Amiens, France,; 3 Eurasian Cancer Research Council, Mumbai, India,; 4 Ateneo de Manila University School of Medicine and Public Health, Quezon City, Philippines,; 5 Saint James School of Medicine, Park Ridge, Illinois, USA,; 6 Department of Family Medicine, Southern Illinois University, Springfield, Illinois, USA,; 7 AMA School of Medicine, Makati, Philippines,; 8 Medisys/Jamaica Hospital Ambulatory Care Center, Jamaica Hospital Medical Center, New York City, New York, USA,; 9 Rutgers Robert Wood Johnson Medical School, New Brunswick, New Jersey, USA,; 10 SUNY Downstate Health Sciences University, Brooklyn, New York, USA

**Keywords:** Rheumatic heart disease, heart defects, congenital, annuloplasty, pediatric cardiology, children operated, developing nation, Chad

## Abstract

**Introduction:**

cardiac valvular diseases (CVDs) are the major cause of cardiovascular morbidity and mortality globally, with predominance of rheumatic heart disease (RHD) in developing countries. Congenital heart defects (CHD) diagnoses are delayed due to socioeconomic factors. This study aims to evaluate the post-operative surgical outcomes of CHD and valvular RHD.

**Methods:**

this study is conducted with 50 patients from Chad, operated on between 2003 and 2012. Post-operative outcomes are evaluated from 2010 to 2012.

**Results:**

with the follow-up of 19 RHD patients who underwent plasty, 8 (42.1%) had no complications, 4 (21%) presented with mild regurgitation, 7 (36.8%) required re-operation due to 6 mitral stenosis (MS) cases (mitral surface range from 0.7 to 1.2 cm^2^) and 1 severe mitral regurgitation (MR) case. While those patients with valve replacement, 2 (50%) had no complications, 1 (25%) had mild regurgitation and 1 (25%) patient died. Two patients with aortic regurgitation (AR) that underwent annuloplasty presented with severe regurgitation. Regarding AR with valve replacement, 3 (60%) had no complications, and 2 (40%) had mild regurgitation. Among the tricuspid regurgitation (TR) patients who had plasty, 6 (85.7%) had no complications, and 1 (14.3%) had severe regurgitation. The surgical repair was curative in all CHD patients. The loss to follow-up rate was 13/50 (26%).

**Conclusion:**

the annuloplasty on rheumatic valve disease (MR and AR) has proven to be disappointing. Plasty is debated without justified indication for AR. The outcomes of CHD, mitral and aortic valve replacement are successful.

## Introduction

Cardiovascular diseases (CVDs) are the leading cause of death globally, with an estimated 17.9 million [1] lives taken each year. CVDs are a group of disorders of the heart and blood vessels and include coronary heart disease, cerebrovascular disease, rheumatic heart disease (RHD), congenital heart disease (CHD) [1]. RHD cases are highest among the world´s disadvantaged population groups. Its prevalence has been rising steadily since 1990, reaching 40.5 million currently affected in 2019 [2].

A seminal study [3] demonstrates that following throat infection by *Streptococcus pyogenes*, the potential role of the cross-reactive antibodies in the development of RHD, by showing that they are able to bind to the endothelial surface, which may lead to inflammation, cellular infiltration and valve scarring. Every year, the RHD claims 291,000 lives worldwide [1]. It also accounts for about 2% of deaths from cardiovascular diseases. Despite it being stopped in several parts of the world, this disease remains prevalent in sub-Saharan Africa, the Middle East, Central and South Asia, the South Pacific, as well as among immigrants and older adults in high-income countries, usually in indigenous peoples [4]. Valvular heart disease due to RHD is very prevalent in sub-Saharan Africa [5]. Currently, the most common treatment for advanced stages of RHD is valve replacement [6], but both mechanical and bioprosthetic valves fail prematurely [7] in these patients due to their young age and limited ability to access and manage anticoagulation treatments [8].

For congenital anomalies, the CHD is the most common, representing a major worldwide health problem. It is a structural abnormality of the heart and great vessels present at birth [9]. A total of 3.12 million babies were born with CHD in 2019 representing 2,305.2 per 100,000 live births, a total of 13.3 million people were living with CHD, and this was the underlying cause of 217,000 deaths, of which 150,000 deaths were in infants <1 year [2]. Although numerous aetiologic investigations have been conducted, only approximately 15% of cases of CHD can be attributable to a known cause [10].

From 2003 to 2012, Chadian patients with heart failure from diverse aetiologies, underwent cardiac surgery, after meeting surgery criteria of the NGO CDE. This study has been conducted in order to focus on the value of mitral annuloplasty rings in young children in a country where the INR (International Normalized Ratio) monitoring for the follow-up of patients, is challenging. For this purpose, we have provided a significant follow-up, for a period of up to ten years, for young cardiac children with diverse CVDs.

## Methods

**Study design and participants:** the study design is mainly a retrospective review of 44 patient profiles. However, an additional 6 patients have been included within the study. Data has been collected on patients with CVD, who received operative treatment in the *Hôpital Européen George Pompidou de Paris (France)*, through the support of the charitable association *Chaîne de l´Espoir* between 2003 and 2012. For the retrospective review, all necessary data in the files of patients operated on before the start of the study period, has been used. For the prospective cohort review, an interview was conducted to collect a case-history of the patients. A data collection sheet was compiled to record the lab results and physical examination. This data collection period spanned from 2010 to 2012.

**Setting:** the study was carried out in the cardiology department of the General National Reference Hospital (HGRN) of N´Djamena, the capital of Chad, and then in the Paediatrics Department of the *Bon Samaritain* Teaching Hospital of Walia, in N´Djaména, Chad. The patients were sent to France and operated on by cardiac surgeons from the charitable association *Chaîne de l´Espoir*.

**The *Chaîne de l´Espoir* programme details:** the *Chaîne de l´Espoir*, an independent and autonomous charitable association started in 1988 under the aegis of *Médecins du Monde*. Under *Chaîne de l´Espoir*, the paediatric cardiology treatment, first transferred to the surgical unit in France, in 1988. Every year, about 80% of the children transferred to France under this programme; overall, 180 to 200 children were transferred during this agreement between *Chaîne de l'Espoir* missions and Chad.

### Characteristics of participants

**Inclusion criteria:** included in this study were: children with cardiac manifestations, between the ages of 1 year and 18; with simple heart disease that is accessible to simple surgical procedure, with a view to definitive cure; heart disease without major operative risk due to past progressive stages or associated pathology.

**Exclusion criteria:** those excluded in this study were: patients under one year (1 year) or over 18 years of age; complex heart disease requiring difficult or dangerous surgery (single ventricle, transpositions of the great vessels) associated with other valve diseases of the left heart; patients with major systemic pulmonary hypertension; patients with an operable cardiac pathology but with high systemic pulmonary hypertension (greater than 100mmHg) and cachectic patients who cannot support the surgical procedure.

### Study variables

**Independent variables:** independent variables include: socio-demographic characteristics (age, sex); weight (kg); height (cm); aetiology of cardiovascular disease; mechanism of cardiovascular disease; surgical technique; valve prosthesis; valve ring; and follow up.

**Outcome details:** the clinical assessment after the surgery is based upon the improvement of physical examination including cough, chest pain, pulse, jugular veins, abdominal palpation for liver-size assessment and hepato-jugular reflux, cardiac and pulmonary auscultation, and blood pressure (mmHg). The dyspnoea was scaled according to the New York Heart Association (NYHA) [11]. The electrical activity of the heart was recorded using an electrocardiogram (ECG) to determine the heart rate, the electrical axis of the heart, cavitary enlargements, bundle branch or atrioventricular blocks, and arrhythmias.

The cause of the valve disease and mechanism of the regurgitation including the dysfunction type (cusp motion abnormality) are described according to the Carpentier´s [12] classification of leaflet motion: Type I normal leaflet motion, Type II excessive motion, and Type III -restrictive motion. Afterwards, a careful assessment was performed of the regurgitant jet by color Doppler, using vena contracta (VC) and proximal isovelocity surface area (PISA). Following two-dimensional (2D) transthoracic echocardiography (TTE), the valve regurgitation is graded mild, moderate or severe [13].

The 2D TTE was performed for confirmation of the diagnosis, quantitation of valve stenosis severity and its consequences, and analysis of valve anatomy. We classified valve stenosis as mild, moderate and severe [14]. We use the standardization for echographic RHD diagnosis consensus criteria of the World Heart Federation [15].

In relation to the congenital heart disease, a three-dimensional echocardiography (3DE) with the same transducers and ultrasound systems as used with adults, but with the addition of high-frequency probes suitable for imaging babies and children, was used in order to do the diagnosis and the assessment of the defect [16].

**Data collection techniques and tools:** the screening of children with heart disease included various stages of processes of sampling. Initial consultation was done with the general practitioner due to significant functional signs; a subsequent plan for consultation with a cardiologist. The second consultation involved clinical examination, ECG, a chest X-ray and an echocardiogram where a fundamental examination was done to determine the aetiology of heart disease, its evolutionary stage, and possible surgical treatment. Once the child has met the criteria, *Chaîne de l´Espoir* was immediately contacted to agree on the treatment plan for the patient and further formalities are conducted. Once consent from the patient's parents is given, and pre-operative fitness is confirmed and an echocardiography and the biological assessment is made in N'Djamena, the surgical procedure is conducted according to the underlying condition of the patient, in France. After the post-operative resuscitation phase of 2 to 3 days, the patient is transferred to a peripheral hospital in France, in the cardiology department. This is followed by a phase of local cardiological monitoring which involves physical examination, clinical and laboratory monitoring of electrocardiogram, the chest X-ray and the transthoracic echocardiography.

**Ethical concerns:** the informed consent of the patient´s legal guardian was obtained both for permission to operate and to use the data for research purposes. They were briefed about the purpose of our research and confidentiality was assured. Before any evacuation for the operation, a parental discharge was read and approved, of which three important paragraphs state:

*“Those responsible for the Chaîne de l´Espoir are authorized to perform any medical or surgical intervention deemed necessary. Any claim for the collection of damages is waived in the event that the child's condition worsens, or any accident occurs during his stay in France. If the patient were unfortunately to die during the stay abroad, repatriation would not proceed at the expense of the Chaîne de l´Espoir.”*

**Statistical analysis:** data collected were entered into an Excel spreadsheet. The results are expressed in terms of number, percentage and in the form of tables and figures.

## Results

**Demographic profile of these operated paediatric cardiology patients:** research participants were predominantly from the age group of 11-15 years of age, representing 23 cases out of 50 study participants (46%). The mean age of the study population was 10.68 years, with the extremes of 1 year and 17 years. The study population has compromised of 31 (62%) female and 19 (38%) male patients, with a sex ratio of 1.63 in favour of female. Yearly trends of surgeries have shown that maximum surgeries were done in 2005 (15), 2007 (11), 2008 (7), 2004 (5), 2010 (4) and 2 cases in 2006, 2009, 2011 while 1 in 2003.

**Aetiology of cardiovascular diseases in operated patients:** out of 50 cases, 38 (76%) had RHD and the remaining 12 patients had CHD (24%). For RHD patients; 22 (57.89%) had mitral regurgitation (MR), five (13.16%) had mixed MR and, three (7.9%) with mixed mitral, aortic, TR. TOF is predominant among congenital ARl cardiac diseases, with 4 out of 11 cases (37%).

**Evaluation of operated patients and immediate surgical procedures and results for those lost to follow-up:** there were 24% (12 cases out of 50) patients not seen in postoperative consultation among those operated on (Table 1). There were patients that require close cardiological consultation among the lost to follow-up patients (Table 1).

**Table 1:** number of patients with rheumatic and congenital heart disease reviewed and not reviewed postoperatively

Rheumatic heart disease										
Etiology	MR, MR + MS	MS	MR	MR + TR	MR + AR	MR + AR + TR	MR + MS + TR		MS + AR	Total
Operated	22	2	1	2	5	3	2		1	38
Reviewed	15	2	1	2	3	3	1		1	28
Not Reviewed	7	0	0	0	2	0	1		0	10
**Congenital heart disease**										
Etiology	TOF	VSD	VSD + ASD	MR	Obstructive cardiomyopathy of RV					Total
Operated	4	3	1	2	1					12
Reviewed	3	2	1	2	0					9
Not reviewed	1	1	0	0	1					3
**Immediate surgical procedures and results for those lost to follow-up**										
Etiology	MR (RHD) n=4	MR + MS (RHD)	MR + MS (RHD)	MR + AR (RHD)	MR + MS (RHD)	MR + TR (RHD) l	MR (CHD)	Obstructive cardiomyopathy of RV		
Surgical technique	CE	Commissurotomy	CE 32	MR (CE 32). AR (Sorin 21)	CE 32	MR (CE 30). TR (CE 26)	Tailor n°25	Plasty, cavo-pulmonary anastomosis		
Immediate outcome	Good	Residual MR	Moderate MR, *Δp=5-6	Good	Moderate MR	Goods	Good	Good		

CE: Carpentier Edwards (used for ring annuloplasty) *Δp: pressure gradient (mmHg) RV: right ventricle

### Functional signs, surgical technique and results of patients operated and reviewed

***Rheumatic mitral regurgitation and mixed mitral disease (mitral regurgitation and mitral stenosis):*** the chest pain, hemoptysis on exertion and syncope on exertion resolved after the operation. Dyspnoea and cough decreased except palpitations, which increased from 4 to 6 cases (Table 2). Mitral replacement surgery and mitral valve replacement have given satisfactory results.

**Table 2:** functional signs, surgical technique and results of patients operated

Rheumatic mitral regurgitation and mitral disease (mitral regurgitation + mitral stenosis)						
Functional signs			Surgical technique (n)	Outcome		
	Pre-operative	Post-operative		Good	Moderate	To operate
Dyspnea	23	7	ring annuloplasty (19)	8	4	7
Cough	14	4				
Chest pain	6	0	Valve replacement (4)	2	1	1 (died)
Palpitation	4	6				
Hemoptysis	3	0				
Syncope	2	0				
**Rheumatic aortic regurgitation**						
**Functional signs**			**Surgical technique (n)**	**Outcome**		
	**Pre-operative**	**Post-operative**		**Good**	**Moderate**	**To operate**
Dyspnea	7	2	annuloplasty (2)	0	0	2
Cough	2	0				
Chest pain	2	0	valve replacement (5)	3	2	0
**Rheumatic mitral stenosis**						
**Functional signs**			**Surgical technique (n)**	**Outcome**		
	**Pre-operative**	**Post-operative**		**Good**	**Moderate**	**To operate**
Dyspnea	4	1	valve replacement (3)	2	0	1
Cough	1	0	Commissurotomy (1)	1	0	0
**Congenital valve disease; 3 mitral regurgitation; 1 aortic regurgitation**						
**Functional signs**			**Surgical technique**	**Outcome**		
	**Pre-operative**	**Post-operative**		**Good**	**Moderate**	**To operate**
Dyspnea	3	0	mitral annuloplasty	2	1	0
Cough	1	0	valve replacement	1	0	0
**Congenital heart disease (shunt and tetralogy of Fallot)**						
**Functional signs**			**Surgical technique**	**Outcome**		
	**Peri-operative**	**Post-operative**		**Good**	**Moderate**	**To operate**
Dyspnea	7	0	communication correction	7	0	0
Cough	1	0				

***Rheumatic aortic regurgitation:*** favourable clinical improvement for dyspnoea, cough and chest pain (Table 2). Patients with aortic plastic surgery must all be re-operated.

***Rheumatic mitral stenosis:*** the surgical techniques (mitral valve replacement and commissurotomy) used in the context of MR have given satisfactory results (Table 2).

***Congenital cardiovascular disease:*** the ring annuloplasty surgery for congenital valve disease is satisfactory. There is a very good operative result in the context of closure of the shunts and tetralogy of Fallot (Table 2).

**The imperfect results of mitral reconstruction:** among 22 patients operated for MR, there were 11 patients with imperfect results; 5 patients with MR after the surgery and 6 patients with mitral stenosis. The imperfect results with residual MI are explained by their starting mechanism (Table 3). All of our patients with MR post-MR surgery increased in height and weight after the operation (Table 3).

**Table 3:** imperfect results for patients operated for MR

Mechanism of MI and prosthetic materials used in patients with significant residual MI after mitral reconstruction						
Age (year)		Mechanism		Ring		Outcomes
9		Dilated ring, A1 prolapse		CE n°28		MR 2/4
9		Annular dilation, valve retraction		CE n°26		MR 3A/4
12		Dilated ring, retraction of the 2 valve leaflets		CE n°26		MR 2/4
11		Commissural fusion, thickening of the large mitral valve		Commissurotomy		MR 2/4
16		Dilated annulus, retraction of the small mitral valve		CE n°30		MR 2/4
**Clinical characteristics and prosthetic materials used in patients with mitral stenosis after MI**						
Age (year)	Time of consultation	Weight (kg) / height (meter)		Ring	Outcomes of stenosis	
		Pre-operative	Post-operative			
7	4 years	17/1,22	32/1,44	CE n°28	MVA=0.7-0.8cm^2^ PG 100mmHg	
9	4 years	27/1,32	32/1,47	CE n°28	MVA=1cm^2^ PG 65mmHg	
10	5 years	23/1,34	35/1,55	CE n°26	MVA=1.2cm^2^ PG 60mmHg	
15	4 years	37/1,45	43/1,51	CE n°30	MVA=1cm^2^ PG 30mmHg	
14	1 year	37/1,45	48/1,65	CE n°34	MVA=1 cm^2^ PG 45mmHg	
13	8 years	48/1,58	54/1,60	CE n°28	MVA=1.1cm^2^ PG 23mmHg	

* PG: peak gradient. MVA: mitral valve area

**Post-operative results of tricuspid regurgitation:** out of the 19 patients with TR that underwent ring annuloplasty, 14 (73.68%) reported to have good outcome, while in five patients (26.32%) regurgitation persisted. Out of these five patients whose regurgitation had leakage in post-surgery, 60% (3) had baseline minor TR and remaining 40% had moderate TR (2). Out of these five cases, three had significant annular dilation, one dilatation and valvular retraction and remaining one rheumatic TR type III. Most of them underwent tricuspid annuloplasty with a ring of Carpentier Edwards (CE) n°26.

**Post-operative results of AR:** out of the 18 patients with AR, 13 had aortic annuloplasty. Out of the 13 patients, 4 had persistent AR, while 1 developed aortic stenosis (AS). All of those who underwent valve replacement did not have any complications post-operatively. Aortic annuloplasty are unable to completely correct aortic leaks: they only temporarily reduce their severity (Table 4).

**Table 4:** rheumatic aortic regurgitation and congenital heart disease outcomes

Rheumatic aortic regurgitation					
Technique	Gesture	Mechanism		Outcomes	
Aortic annuloplasty	Commissurotomy, sigmoid extension	Commissural fusion		AR 2/4, AS 1.5	
	Extension of the 3 leaflets	Retraction of the valve leaflets		AR 2/4, AS 1.5	
Valve replacement	Sorin n°23	Sigmoid retraction		Good	
	Carbomedics n°25	Retraction, thickening, sigmoid fibrosis		Good	
	Carbomedics n°21	valve vegetation, sigmoid rupture		Good	
	Sorin n°21	Thickening and retraction of the valve leaflets		Good	
	Sorin n°21	Thickening and retraction of the valve leaflets		Good	
**Congenital heart disease**					
**Type (number)**	**Mechanism**		**Surgical technique**		**Outcomes**
Tetralogy of Fallot (4)	Subaortic VSD, dextrocardia		Correction		Good
VSD (3)	VSD		Correction		Good
VSD + ASD (1)	VSD + patent foramen ovale		Correction		Good
RV Obstructive cardiomyopathy (1)	Distended tricuspid ring, anterior leaflet hypoplasia, moderator band hypertrophy		Band incision, ring		Not reviewed
MR	Dystrophic annular dilation		mitral annuloplasty ring		Good
MR	Annular dilation, A2 dislocation		mitral annuloplasty ring		Good
MR + AR	Mitral: annular dilation, A2 prolapse.		mitral annuloplasty ring		MR 2/4
	Aortic: coronary sigmoid prolapse		Aortic:sigmoid fold		AR 4/4

**Congenital heart disease outcome:** when reviewing the profile of operated CHD, the majority had TOF (4), followed by VSD (4), MR (3) and 1 case of RV obstructive cardiomyopathy, as shown in the table below. Tetralogy of Fallot (TOF) and ventricular septal defect (VSD) underwent correction surgery, while remaining vulvar diseases underwent plasty with good outcome (Table 4).

**Summary of retained and operated patients:** out of 50 operated patients, 38 (76%) had post-operative consultation. Almost 42% (21 out of 50) reported good outcomes, 8 (16%) patients defaulted, while 7 (14%) patients died after surgery. Of all our operated patients, 16% were lost to follow-up. Out of seven deaths, four (57.14%) have a baseline history of CHDs and the remaining three RHD (42.86%). Out of the four CHD patients, MR was the most common aetiology (4/4) with the added effect of TR in one patient. While for RHD patients, rheumatic MR was common to all patients with the added aetiology of AR in two cases and TR in one case.

## Discussion

This hospital-based study was conducted on 50 Chadian patients aged 1 to 17 years with heart disease registered and retained from 2003 to 2012 at the General National Reference Hospital (43 patients) and at the *Bon Samaritain* University Hospital of Walia (7 patients) in N’Djamena, to evaluate the post-operative surgical outcomes after the management of valvular abnormalities in RHD and CHD. Most of these patients were from outside N’Djamena, and were referred to the capital for adequate care with or without a diagnosis of evoked heart disease.

**Demographic profile of these operated paediatric cardiology patients:** the study showed that among the 50 children operated on, the female sex dominated with 62% of cases (37 females, 22 males) with a sex ratio of 1.63 in favour of the female sex. Out of 50 paediatric patients who underwent the surgery, 46% (23 cases) aged between 11 and 15 years old. The high frequency of paediatric cardiology patients of this age-group in this study population could be explained possibly because of their capacity to express functional symptoms at this age. It is also explained by the age limit required by the *Chaîne de l´Espoir*. Most of the children (15/50) were operated on in 2005. This high rate of performed surgeries in the specific year might be explained by the availability of staff to prepare clinical and administrative files for the evacuation of cardiac patients who can be operated on in France.

**Aetiology of cardiovascular diseases in operated patients:** these 50 operated heart disease patients were classified into ¾ of rheumatic heart disease (76%, 38) and remaining ¼ in CHD (24%, 12). Of significance here, is the point raised above that, there has been a decrease of RHD in industrialized countries. RHD however, still remains a scourge in sub-Saharan Africa. The trend of the causes of acquired heart valve disease in Western countries has been characterized by a gradual decrease in the frequency of rheumatic valve disease and the increase in the frequency of dystrophic and degenerative causes in Western countries [17]. This is possibly due to the increase in life-expectancy and the decrease in the incidence of RHD. In 2015, Oceania, South Asia, and central sub-Saharan Africa were observed as having the highest age-standardized mortality, due to the prevalence of rheumatic heart disease [18]. These high results of death due to RHD could be explained by the high frequency of RHD as a major aetiological factor of these valve diseases.

**Rheumatic heart disease:** in our study, mitral valve damage was the most common. It was most often MR, but also mitral disease (mitral regurgitation + mitral stenosis) in 3 patients. This is the same as the literature that found the estimation of 50 to 60% cases of mitral valve damage in chronic rheumatic heart disease [19]. After the mitral valve, the aortic valve was the most affected, with 20% of our patients with AR. In young patients, MR is the predominant heart injury, aortic regurgitation is less frequent, and the frequency of mitral stenosis would gradually decrease from childhood to adulthood [20]. For tricuspid valve involvement, in the 24 cases of TR, it was always multiple valves affected, comprising 18 cases of functional TR and 6 cases of rheumatic TR. Tricuspid involvement is the rarest if we consider only organic TR, marked by retractile fibrosis of one or more leaflets of the valve. However, it comes in second position, immediately behind mitral valve, if we take into account all functional TR, 3 times more frequent than organic TR and more than half of which requires corrective annuloplasty to avoid the persistence of irreversible RV heart failure signs. Tricuspid valve involvement is the rarest of all valve diseases; it is most often associated or secondary to left valve involvement [21]. The prevalence of significant tricuspid involvement in rheumatic disease is 14% in our study.

**Congenital heart disease *:*** out of the 12 congenital heart diseases reported in our study, TOF predominated with four cases (34%) followed by VSD with (25%) then VSD and ASD one (8%) case. The literature [22] rather reveals the predominance of VSD, over ASD and tetralogy of Fallot. By only accepting children over one year of age, we let die all transposition of the great vessels which are great emergencies, but above all a considerable number of large VSDs which evolve in a few months towards the great irreversible pulmonary hypertension. This explains the low representation of VSD in our study.

**Outcome of mitral valve surgery:** eight (8) cases out of 19 (42%) of good results were found. The results of the mitral annuloplasty ring are mostly satisfactory. Among the imperfect results, there are 30% (6 out of 20) of mitral stenosis, 20% (4 out of 20) of persistent leakage, 5% (1 in 20 cases) each of a combination of leakage + stenosis, of significant persistent leakage, and congestive heart failure from hypokinetic LV failure. The patient with congestive heart failure due to hypokinetic LV failure died at the *Bon Samaritain* hospital 80 days after the surgery. It therefore appears that mitral annuloplasty ring is a palliative surgery and not curative; it is subject to the progressive inflammatory risk of rheumatic fever which remains all the more important, as the patient is younger and from a developing nation, or among disadvantaged populations within developed nations [23]. There is also the risk of constitution of relative mitral stenosis by inextensibility of the annuloplasty during years of growth. This relates to studies of Kwan *et al*. [24]: “Failures can be explained by anatomical lesions caused by rheumatic fever, by uncorrected mitral valve ring dilation, persistent valve prolapse, valve and subvalvular damage”.

**Clinical features and prosthetic materials used in patients with residual MI after mitral reconstruction *:*** the pre-operative MR mechanisms included large dilation of the mitral ring, retraction of the mitral valve leaflets, commissural fusion, and thickening of the mitral valve. These cited mechanisms explain the imperfection of the surgical results.

**Clinical features and prosthetic materials used in patients with mitral stenosis after MR *:*** the patients with mitral stenosis after mitral annuloplasty ring for MR all increased in height and weight after the surgery. Their postoperative evaluation time varies for each patient from 1 to 8 years. The increase in cardiac output, proportional to the considerable weight and height gain and the lack of mitral annulus area growth that accounts for the development of progressive mitral stenosis: this is like a mismatch between cardiac output and mitral surface.

**Outcomes of rheumatic aortic valve surgery:** there is no aortic stenosis, only aortic regurgitation. Of the 2 aortic annuloplasty performed, there is a persistence of aortic leakage and an association of leakage and stenosis of the aortic valve. The aortic annuloplasty surgery was performed by using a patch extension of the autologous pericardium. This would explain the disappointing results because this technique can only be performed successfully in a bicuspid aortic valve.

**Outcome of tricuspid valve surgery:** for all TR (13 functional and 6 rheumatic) reviewed, ring annuloplasty was the technique performed with 14 (73.68%) out of 19 cases of good results and 5 (26.32%) out of 19 of persistent tricuspid leakage. Of the 5 residual tricuspid regurgitation, 3 are minor and 2 are moderate. Tricuspid annuloplasty is a good intervention if it is a functional TR by simple annular dilation. On the other hand, it is not sufficient in the case of organic rheumatic tricuspid affection which involves retraction of the valve leaflets and chordae tendineae and the commissural fusion: this requires more surgical manoeuvre (partial commissurotomies, release of chordae tendineae) to obtain a good result. The use of tricuspid mechanical prostheses is definitely abandoned due to the high number of valve thrombosis and sudden death. When a patient is at NYHA stage IV with a proven right ventricular state massive edema of clinical and biological hepatic insufficiency, the operative mortality rate is very high. The late mortality rate is also very high if the RV dysfunction persists, that is, if the intervention is too late to allow recovery of the RV [25]. Rheumatic Tricuspid valve repair, in the same way, the rheumatic mitral valve repair, is not a definitive solution if there is not simultaneously rigorous rheumatic fever prevention.

**Summary of patients retained and operated on:** fifty (50) patients benefited from cardiac surgery in France, and returned to Chad. Seventy-six percent (76%) (i.e. 38 out of 50 patients) of cases were reviewed in postoperative consultation. Among the patients reviewed, we found 42% of the cases (21 out of 50 patients) with good results, 16% of the cases (8 out of 50 patients) to be operated again and 12% of the cases (6 out of 50 patients) to be followed up regularly. There were also 16% of cases (8 out of 50 patients) lost to follow-up, and 14% of cases (7 out of 50 patients) of death after the operation.

**Limitations:** as our study is both retrospective and prospective, we were not able to collect all the data pre-established in our data collection sheets. The patients had been evaluated by various physicians from different hospitals previously, over the years before they were sent to France for cardiac surgery. Additionally, there were financial difficulties in relation to resources, as well as the functioning of the health system, to systematically carry out a certain number of assessments of patients seen in post-operative consultation conditions which frustrate the follow-up. This is why some data could not be collected even though it appeared in the data collection sheets. Among these data, we can cite the anthropometric parameters, the NYHA classification, the results of the chest X-ray, and the examinations carried out in the laboratory.

## Conclusion

Our study found, that one third of ultimate reoperations after mitral annuloplasty ring for rheumatic MR, are due to the progressive onset of symptomatic tight MS. This occurs more frequently when the annuloplasty ring lasts and the patient gains weight and increases in height. This stenosis results from the mismatch between the annuloplasty ring in place and the oxygen requirement with the patient´s growth. Aortic annuloplasty for rheumatic aortic regurgitation by using a patch extension of the autologous pericardium has produced disappointing results. Regarding the tricuspid valve repair, in 14 out of 19 cases, they proved to be remarkably effective, so long as there was no significant pulmonary hypertension or significant annular dilation. There is a remarkable success of surgical procedures for well-selected CHD: VSD, ASD, and tetralogy of Fallot. The large number of lost to follow-up cases (8 cases out of 50) highlights the great difficulty in monitoring operated patients, due to the remoteness and dispersal of the patients, and the patients´ incomprehension of their affections that required the strict necessity of regular follow-up.

### What is known about this topic

- Prevalence of rheumatic fever in developing countries;
- First rank of the mitral valve affection in rheumatic disease among other cardiac valves;
- Good result of mitral valve prosthesis in rheumatic valve disease.

### What this study adds

- The result of mitral annuloplasty ring due to RHD in children;
- The relationship of the growth of children that underwent mitral annuloplasty ring and the development of mitral stenosis after rheumatic mitral valve repair;
- The disappointing result of aortic annuloplasty using a patch extension of the autologous pericardium for large rheumatic aortic regurgitation in children.

## References

[1] World Health Organization (WHO) Cardiovascular diseases (CVDs).

[2] Roth GA, Mensah GA, Johnson CO, Addolorato G, Ammirati E, Baddour LM (2020). Global Burden of Cardiovascular Diseases and Risk Factors, 1990-2019: Update From the GBD 2019 Study. J Am Coll Cardiol.

[3] Galvin JE, Hemric ME, Ward K, Cunningham MW (2000). Cytotoxic mAb from rheumatic carditis recognizes heart valves and laminin. J Clin Invest.

[4] World Health Organization (WHO) Rheumatic heart disease.

[5] Carapetis JR, Steer AC, Mulholland EK, Weber M (2005). The global burden of group A streptococcal diseases. Lancet Infect Dis.

[6] Zilla P, Brink J, Human P, Bezuidenhout D (2007). Prosthetic heart valves: catering for the few. Biomaterials.

[7] Hammermeister K, Sethi GK, Henderson WG, Grover FL, Oprian FL, Rahimtoola S (2000). Outcomes 15 years after valve replacement with a mechanical versus a bioprosthetic valve: final report of the Veterans Affairs randomized trial. J Am Coll Cardiol.

[8] Pelajo CF, Lopez-Benitez JM, Torres JM, de Oliveira SK (2010). Adherence to secondary prophylaxis and disease recurrence in 536 brazilian children with rheumatic fever. Pediatr Rheumatol Online J.

[9] Mitchell SC, Korones SB, Berendes HW (1971). Congenital heart disease in 56,109 births. Incidence and natural history. Circulation.

[10] Van der Bom T, Zomer AC, Zwinderman AH, Meijboom FJ, Bouma BJ, Mulder BJ (2011). The changing epidemiology of congenital heart disease. Nat Rev Cardiol.

[11] The Criteria Committee of the New York Heart Association (1994). Nomenclature and Criteria for Diagnosis of Diseases of the Heart and Great Vessels.

[12] Carpentier A, Chauvaud S, Fabiani JN, Deloche A, Relland J, Lessana A (1980). Reconstructive surgery of mitral valve incompetence: ten-year appraisal. J Thorac Cardiovasc Surg.

[13] Lancellotti P, Tribouilloy C, Hagendorff A, Popescu BA, Edvardsen T, Pierard LA (2013). Recommendations for the echocardiographic assessment of native valvular regurgitation: an executive summary from the European Association of Cardiovascular Imaging. Eur Heart J Cardiovasc Imaging.

[14] Baumgartner H, Hung J, Bermejo J, Chambers JB, Evangelista A, Griffin BP (2009). Echocardiographic assessment of valve stenosis: EAE/ASE recommendations for clinical practice. Eur J Echocardiogr.

[15] Remenyi B, Wilson N, Steer A, Ferreira B, Kado J, Kumar K (2012). World Heart Federation criteria for echocardiographic diagnosis of rheumatic heart disease-an evidence-based guideline. Nat Rev Cardiol.

[16] Simpson J, Lopez L, Acar P, Friedberg MK, Khoo NS, Ko HH (2017). Three-dimensional echocardiography in congenital heart disease: an expert consensus document from the European Association of Cardiovascular Imaging and the American Society of Echocardiography. J Am Soc Echocardiogr.

[17] d'Arcy JL, Coffey S, Loudon MA, Kennedy A, Pearson-Stuttard J, Birks J (2016). Large-scale community echocardiographic screening reveals a major burden of undiagnosed valvular heart disease in older people: the OxVALVE Population Cohort Study. Eur Heart J.

[18] Watkins DA, Johnson CO, Colquhoun SM, Karthikeyan G, Beaton A, Bukhman G (2017). Global, Regional, and National Burden of Rheumatic Heart Disease, 1990-2015. N Engl J Med.

[19] Dass C, Kanmanthareddy A Rheumatic Heart Disease.

[20] Moore AG, Murphy JG, Lloyd MA (2007). Rheumatic heart disease. Mayo Clinic Cardiology - concise Textbook.

[21] Dreyfus GD, Martin RP, Chan KM, Dulguerov F, Alexandrescu C (2015). Functional tricuspid regurgitation: a need to revise our understanding. J Am Coll Cardiol.

[22] Pavlicek J, Gruszka T, Kapralova S, Prochazka M, Silhanova E, Kaniova R (2019). Associations between congenital heart defects and genetic and morphological anomalies. The importance of prenatal screening. Biomed Pap Med Fac Univ Palacky Olomouc Czech Repub.

[23] Department of Child and Adolescent Health and Development World Health Organization The Current Evidence for the Burden of Group A Streptococcal Diseases.

[24] Chan KL, Chen SY, Chan V, Hay K, Mesana T, Lam BK (2013). Functional significance of elevated mitral gradients after repair for degenerative mitral regurgitation. Circ Cardiovasc Imaging.

[25] Sarralde JA, Bernal JM, Llorca J, Pontón A, Diez-Solorzano L, Giménez-Rico JR (2010). Repair of rheumatic tricuspid valve disease: predictors of very long-term mortality and reoperation. Ann Thorac Surg.

